# Skills and epistemic cultures in artificial intelligence research: evidence from job postings

**DOI:** 10.3389/fsoc.2025.1655903

**Published:** 2025-10-10

**Authors:** Anamaria Nastasa, Monica Mihaela Maer Matei, Cosima Rughiniş, Dinu Ţurcanu

**Affiliations:** ^1^Department of Education, Training and Labour Market, National Scientific Research Institute for Labor and Social Protection (INCSMPS), Bucharest, Romania; ^2^Department of Economic Informatics and Cybernetics, Bucharest University of Economic Studies, Bucharest, Romania; ^3^Faculty of Sociology and Social Work, University of Bucharest, Bucharest, Romania; ^4^Faculty of Electronics and Telecommunications and National Institute of Innovations in Cybersecurity “CYBERCOR”, Technical University of Moldova, Chişinău, Moldova

**Keywords:** text mining, skills, artificial intelligence, researchers, epistemic cultures, skills needs

## Abstract

Artificial Intelligence (AI) has begun to transform the labor market, allowing technologies to perform some of the tasks previously performed only by humans. Previous studies have shown that artificial intelligence technologies have reshaped workplaces and tasks structures, generating new skill demands in the labor market. However, there is limited research on how the required skills and underlying epistemic orientations of AI-related academic jobs are communicated during the hiring process. The present study explores this discursive construction of the researcher role by analyzing the skills and competencies prioritized in AI-related academic job postings. To achieve the study's goals, we used data on job descriptions from around 800 jobs posted on the EURAXESS platform until January 2024 using descriptive text mining methods and Latent Dirichlet Allocation (LDA) topic modeling. The findings revealed a strong demand for research and digital skills, as well as career development, communication, mobility, and enterprise skills. The results also reveal seven distinct thematic clusters, which we interpret as representations of different epistemic cultures being signaled to prospective candidates. The results can be valuable for policymakers, research institutions, and universities.

## 1 Introduction

Artificial intelligence (AI) has begun to transform the economy, enabling machines to perform some of the cognitive tasks previously performed by humans. Over the next decade, many existing tasks will be replaced by machines while new ones will emerge (Brynjolfsson et al., 2019). Consequently, it has become important to identify those specific skills for which technological change creates demand. Digital technologies reshape skill demands while also enabling researchers to analyze large datasets through natural language processing techniques. In this way, we can link information on technologies with information on skills to discover how the content of existing jobs is changing.

While the creation of new roles and higher efficiency are key features of this transformation, a more critical perspective also highlights concerns about the potential for these technologies to exacerbate job precarity and contribute to the deskilling of certain professions (Ponce Del Castillo, 2024). Job precarity refers to employment characterized by instability, insecurity, low wages, and limited social protection, often lacking traditional employee benefits and rights. Recent studies highlight that incorporating AI into the workplace can increase stress and discrimination and is often linked by employees to job insecurity and psychological distress (Lane and Saint-Martin, 2021; Presbitero and Teng-Calleja, 2023). Moreover, adoption of these technologies is susceptible to intensifying structural inequalities in the labor market, often associated with job precariousness (Broady et al., 2025; Ponce Del Castillo, 2024). In contrast to the first insights from Cedefop's AI skills survey (Cedefop, 2025), which stress the need for upskilling and reskilling as key components of effective AI adoption strategies, researchers are concerned about the deskilling associated with these technological advancements. This is a phenomenon where workers lose proficiency in certain skills because AI now performs those tasks, leading to skill erosion. The studies draw attention to the fact that although we talk about human-AI augmentation and not substitution, the phenomenon of deskilling can occur (Huseynova, 2024). However, understanding how AI integration affects skill requirements requires consideration of the nature of the tasks involved (Crowston and Bolici, 2025).

Therefore, AI is associated with a redefinition of roles and tasks, leading to a less predictable restructuring of the labor market with heterogeneous effects across industries and occupations. All these concerns also extend to the research landscape. Integrating AI as a research tool raises important challenges, such as the stability of academic skills and jobs, reducing professional skills development, and deskilling (Chubb et al., 2022). While AI offers significant benefits to research and researchers, its negative consequences call for critical attention. Investigating this phenomenon from diverse perspectives is essential to developing a comprehensive understanding of AI's impact on scholarly practice. Our research aims to contribute to the scarce literature on AI's impact on research practices.

Epistemic culture is an important concept in the literature regarding the scientific process and organization. (Knorr-Cetina 1999) and (Knorr-Cetina 2007) argues that science is not a monolith but multiple strands of epistemic diversity. According to (Knorr-Cetina 2007), epistemic cultures are “those sets of practices, arrangements, and mechanisms bound together by necessity, affinity and historical coincidence which, in a given area of professional expertise, make up how we know what we know” (p. 363). She argues that these cultures create and warrant knowledge (Knorr-Cetina, 1999). This focus on the “machinery” of knowledge creation is a cornerstone of the broader field of the sociology of scientific knowledge (SSK) and practice-oriented studies of science. This perspective moves beyond analyzing scientific theories as abstract products and instead investigates the research communities' day-to-day, socially and materially embedded practices of research communities. For instance, Karin Knorr-Cetina's work on “the manufacture of knowledge” demonstrates how scientific facts are constructed within the contingent, localized environment of the laboratory, rather than being simple reflections of nature (Knorr-Cetina, 1981). Similarly, scholars like (Pickering 1995) emphasize the “mangle of practice,” a dynamic interplay where human intentions and material agency, such as instruments, or in our case, algorithms and datasets, are mutually shaped over time. Adopting this perspective allows us to see AI-related research not as a monolithic enterprise, but as a collection of emerging epistemic cultures, each with its own distinct set of legitimized practices, tools, and community norms, which are articulated and reproduced through documents like job postings. Some authors also state that even within the same discipline, multiple epistemic cultures are observed in how scientific output is legitimized in the process of science production (Keller and Poferl, 2016).

Epistemic cultures thus refer to the specific sets of practices, values, methodologies, and social mechanisms through which knowledge is created and validated within particular communities or fields. The concept is relevant in AI-related research, because different epistemic cultures emphasize distinct sets of competencies and skills, depending on their disciplinary approaches, methodological preferences, and goals. For instance, a strongly mathematical and computational epistemic culture prioritizes technical proficiency and analytical skills, whereas interdisciplinary epistemic cultures stress communicative competencies, collaborative abilities, and broader ethical or societal considerations. Thus, analyzing skill requirements through the lens of epistemic cultures allows us to clearly identify and understand how different research communities within AI define and prioritize the competencies necessary for scientific work.

On the other hand, the epistemic culture might also play a crucial role in constructing, teaching, and understanding skills. Researchers observed that some competencies, such as generic skills, are understood differently depending on the domain. For example, (Jones 2009) showed that communication, critical thinking, analytic skills, and problem-solving are conceptualized and taught differently depending on the disciplinary focus. Similarly, the same author showed differences in how disciplines understand critical thinking in economics and history (Jones, 2007). (Brew 2008) argues that under the contemporary changes and developments where new disciplines are arising, the rigidity of domains with fixed demarcations/boundaries among disciplines must be contested, accepting more fluid concepts of interdisciplinarity. He advocates for a more fluid understanding of interdisciplinarity and draws ideas from Bauman's (2006) concept of “liquid modernity,” which describes societies that change rapidly, preventing habits and routines from becoming institutionalized (Brew, 2008). In this context, disciplinary changes are increasingly manifest as forms of interdisciplinarity.

Some authors argue that some Artificial Intelligence epistemic cultures are constructed or built through online communities and adjacent activities such as forums, job boards, blogs, conferences, competitions, forecasting, and other online activities (Ahmed et al., 2024). While the studies above outline the general competencies for researchers, our analysis treats job advertisements as rich discursive artifacts. From a sociological perspective, these documents are more than functional lists; they are public declarations that construct and legitimize the very identity of the sought-after researcher. We draw inspiration from C. Wright Mills' concept of “vocabularies of motive,” which posits that our stated intentions are socially learned justifications for our actions (Mills, 1940). In this light, a job posting provides the accepted vocabulary for a field: it tells prospective candidates the socially approved skills, goals, and motivations are doing valuable work within that specific research community. Furthermore, following (Scott and Lyman 1968), we can understand a job posting as a form of public “account,” a statement made by an institution to explain and justify the necessity of a role and its specific requirements. By analyzing these vocabularies and accounts, we can investigate how a field like AI discursively negotiates its boundaries, prioritizes certain competencies, and legitimizes particular forms of knowledge and practice.

In this sense, jobs posted through research boards such as EURAXESS help to form and, at the same time, maintain the community while simultaneously promoting the present projects and interests of research groups/researchers. Skills required for a scientific position are an implicit part of the epistemic culture in a specific field; firstly, these skills are tied to methodological and theoretical aspects of a discipline. Moreover, skills are often formally and implicitly transmitted in specific research settings. On the other hand, through hiring, the expectations from both the organization and the scientific community are implicit. At the same time, Artificial Intelligence is not a singular community. Thus, depending on the disciplinary focus, there is a probability that AI has multiple epistemic cultures.

Building on this framework, the present research uses job postings not as a direct measure of on-the-ground practices, but as important discursive artifacts that show how research communities present themselves and define the 'ideal' candidate. Therefore, this study aims to explore how the role of the researcher in AI-related fields is constructed through the language of skill requirements, providing an overview of the competencies and thematic focuses being prioritized in the academic hiring landscape. This approach allows us to investigate the implicit boundaries and values of emerging epistemic cultures within AI as they are communicated to the next generation of researchers.

In the next section, we review the general literature on the skills required of researchers, followed by a discussion of the epistemic cultures within AI-related research. We then review the literature on Artificial Intelligence, and the associated skill demands in the labor market. The literature section is followed by the methodology section, which describes the data and analytical approach, the presentation of results, and finally the conclusions and discussion.

## 2 Literature review

### 2.1 Required skills for researchers

Several research studies examined the skills of researchers in different domains and stages. (Maer-Matei et al. 2019), (Durette et al. 2014), and (Mantai and Marrone 2022) studied the skills needs of early career researchers. (Mantai and Marrone 2022) showed that transferable skills are important for hiring PhD students. Among these most desired skills are digital, communication, cognitive, interpersonal, and personal attributes (Mantai and Marrone, 2022). (Maer-Matei et al. 2019), on the other hand, showed a more granular view indicating the salience of data processing and analysis skills, as well as teaching skills and management skills (time, risks, projects) for early career researchers in mathematics, computer science, engineering, and economics. (Durette et al. 2014), on the other hand, identified six general types of competencies in PhD students such as knowledge and specialized technical skills, transferable skills that can be formalized (communication, management, language skills, commercial skills etc.), transferable skills that cannot be formalized (cognitive skills, collaboration, leadership, innovation attributes), dispositions (creativity, rigor), behaviors (curiosity, patience, honesty etc.), meta-competencies (learning and adaptation capacities). One important framework some authors use when studying early career researchers' skills is the EURODOC report proposed by (Weber et al. 2018). (Weber et al. 2018) grouped nine types of skills: (1) career development skills; (2) cognitive skills; (3) communication skills; (4) digital skills; (5) enterprise skills; (6) interpersonal skills; (7) mobility (intersectoral and international); (8) research skills; and (9) teaching and supervision skills. This taxonomy was also used to unveil insights about researchers at all career stages. In this sense, (Mantai and Marrone 2023) observed that research skills are the most important in the career progression of researchers, followed by teaching and supervision skills. Other skills that have gained importance over the last few years are those related to enterprise skills (or marketization; Mantai and Marrone, 2023). We used Weber et al.'s (2018) EURODOC framework to group the skills for the interpretation.

While these studies provide important insights regarding skills requirements in academia in different domains, there are not any studies on Artificial Intelligence-related researchers. The present study completes this gap, providing an exploratory overview of the skills required in this emergent field and associated fields. While these general researcher skills are important across disciplines, their manifestation can vary significantly depending on the epistemic culture of the field. In the case of AI-related research, understanding these cultural differences is important in interpreting skill requirements.

### 2.2 The epistemic cultures of AI-related research

The structure and priorities of AI-related research can be understood through the theoretical lenses of sociotechnical imaginaries and the competition for scientific capital (Rosner, 2025). National and institutional research agendas are shaped by “sociotechnical imaginaries,” socially shared visions of desirable futures that guide technological development and policy priorities. These imaginaries help explain why certain research areas, such as sustainability-aligned AI in Europe vs. state-led industrial applications in China, gain prominence (Rosner, 2025). Concurrently, the global research landscape operates as a competitive scientific arena where institutions in high-income countries, which dominate AI-related research output for “scientific capital,” prestige, visibility, and influence (Obreja et al., 2024; Rosner, 2025). This competitive environment, which is heavily determined by economic development, drives the strategic recruitment of talent and the formation of specialized research clusters (Rosner, 2025).

Such studies offer a macro-level context for interpreting the skills and epistemic cultures reflected in academic job postings. The mapping of the AI-related research landscape, with its distinct thematic dimensions, uneven disciplinary integration, and geographic concentration, shows that the demand for specific skills is not arbitrary but is a direct reflection of these broader structural dynamics (Rosner, 2025). The thematic clusters identified in job advertisements can be seen as micro-level manifestations of dominant sociotechnical imaginaries and institutional strategies for accumulating scientific capital in a rapidly evolving and highly competitive global field (idem). Understanding this landscape is crucial for policymakers and institutions aiming to cultivate research ecosystems that are not only technologically innovative but also ethically responsible and socially integrated (Obreja et al., 2025; Rosner, 2025).

Although the literature provides limited evidence on the AI-related skills of researchers, it does contain literature findings on the impact of AI and its associated skill requirements in the labor market, which can provide an overview of the overall changing labor market landscape.

### 2.3 Artificial intelligence and skill requirements

In this subsection, we provide an overview of the literature regarding the impact of artificial intelligence on job vacancies, looking at research using text-mining techniques. In developing the research methodology, we started with the most recent results identified in scientific literature. The criteria for the selection of references were the following: (i) the investigation should be based on text analysis, (ii) the data analyzed should represent the descriptions in job vacancy advertisements, (iii) the research objectives should be aimed at the impact of artificial intelligence or digitalization on the demand for skills.

The general 21st-century skills needed to attain labor market success, regardless of the industry, were revealed by an analysis of job vacancy advertisements for 2017. Top among these were: written and oral communication, collaboration, and problem solving (Rios et al., 2020). These foundational skills soon evolved to meet specialized technological demands as Industry 4.0 transformed labor markets. For example, an analysis of Industry 4.0-related job vacancies used text mining on LinkedIn advertisements (April–July 2018), identifying two clusters of job profiles. The first was exclusively focused on Industry 4.0-related knowledge: cyber systems and the internet of things for robotic manufacturing; the second group of job profiles was focused on more general knowledge tailored to Industry 4.0: supply management, customer satisfaction, and enterprise software. The skills required by the labor market were related to the following areas: project management, machine learning, big data, computer science and data analytics (Pejic-Bach et al., 2020). Similarly, (De Mauro et al. 2018) focused on big data professions. They identified five types of skills through text-mining techniques: cloud skills, coding skills, database management, data architecture skills, project management skills, systems management skills, distributed computing skills, data analytics skills, and business impact skills. Moreover, they identified two types of job families related to big data: technology-enabler professionals and business-impacting professionals. This focus on technical-functional and business-organizational competencies was reinforced by (Persaud 2020), who examined the skills needed for positions in big data analytics. The text analysis of the advertisements for these positions yielded four broad categories of knowledge, skills, and abilities that employers seek for big data-related professions. These are data analytics, informatics, business, and soft skills. The first two focus on technical knowledge and functional and cognitive skills, while the latter two focus on organizational, business, and management skills and personality traits, which fall under social competencies and meta-skills (Persaud, 2020).

This specialization accelerated as AI adoption reshaped skill demands across industries. An investigation of AI-related job postings across 14 countries from 2019 to 2022 revealed the heterogeneity of AI skills demanded depending on industry or occupation. The analysis showed that AI-related jobs are concentrated in the fields of professional services, ICT, and manufacturing. However, the Professional's major group included the highest share of online vacancies demanding AI-related skills. For that specific period, employers' requirements predominantly include skills related to machine learning. Technical skills related to big data, business intelligence, and cloud computing also differentiate AI employers from others. Moreover, skills related to innovation, problem-solving, or ethical standards are essential for this domain (Borgonovi et al., 2023). Similar research was undertaken for the US economy before 2019, showing that Software and Cognitive skills are mostly associated with AI-related jobs (Alekseeva et al., 2021).

Subsequently, these skill requirements in AI-related jobs began transforming again with the emergence of generative AI. In the last year, the discussion related to the impact the digitization on skills demand has focused on the changes brought about by the use of generative artificial intelligence in performing different tasks (Giordano et al., 2024). The complexity of these AI models requires the acquisition of knowledge and skills in order to reach competent use. The absence of such expertise can be a risk that can turn ChatGPT tools into a hindrance rather than a solution for radical change. Consequently, understanding the technical potential and limitations of AI tools and acquiring proficiency in their use is critical for the future of artificial intelligence. Understanding the skills affected by ChatGPT can provide a clearer picture of how the labor market will develop in the near future. Studies based on analyzing the tasks for which users request ChatGPT have provided preliminary results in this direction. The study concluded that this tool has an impact on programming skills. Consequently, future skills that developers should acquire in order to effectively use these new technologies to write code and remain competitive in the labor market will need to be identified. In terms of humanities, ChatGPT is expected to have an impact on writing-related skills, given its text-generation capabilities. Research has shown that users frequently request ChatGPT for tasks related to writing a script, rewriting articles, and creating online news content. ChatGPT can improve writing skills by influencing both form and content. Some of the skills affected by ChatGPT are at the intersection of STEM and the humanities. These skills, such as digital content creation or problem-solving (that incorporate both STEM and humanities particularities), demonstrate the potential of ChatGPT to bridge the gap between these traditionally distinct fields (Giordano et al., 2024).

These studies show, on one hand, the use of text-mining approaches for identifying evolving skill demands within the context of recent technological change, including AI adoption and generative AI. On the other hand, these studies emphasize technical and non-technical skills that emerge in different industries as AI technologies emerge and further develop. Building on these studies, in the following section, we present this paper's methodological approach. It involves a similar text-analysis approach to examine AI-related research positions advertised on EURAXESS, focusing on their associated skill requirements.

## 3 Materials and methods

While previous studies offer valuable insights into AI's impact on skills, they do not address the academic research sector, where numerous innovations in AI have emerged. This study aims to address this gap by examining AI-related research positions and their associated skills requirements and by providing up-to-date insights into how technological advancements are shaping expertise within the research sector. Building on these previous findings, the present research aimed to examine the skills needed among researchers working in artificial intelligence, related fields using artificial intelligence, or fields that have artificial intelligence as a thematic area of research projects.

### 3.1 Data collection pre-processing

The data analysis is based on job advertisements published until January 5, 2024, on the EURAXESS platform. EURAXESS is a European Union platform where research vacancies are posted, and information on living/working in Europe, research career development, and information on European research initiatives can be found. A job advertisement posted on this site generally includes the following fields: research organization, country, research field, researcher profile, and job description. In addition, the advertisements also contain information on requirements, location, additional information, contact details, and the application process. Only the text from the offer description and requirements where available (including subfields on skills/qualification, specific requirements) was analyzed.

For this study, only jobs whose content included the exact combination of words “Artificial Intelligence” were collected. This approach captures both positions directly in AI and others where AI is a thematic keyword, rather than a core research area. No separate classification between these categories was performed. This limitation was partly due to constraints in the EURAXESS search functionality, which does not allow advanced query options such as filtering by the context in which a term appears (e.g., as a primary research field vs. a peripheral mention). The search engine retrieves postings based on keyword matches anywhere in the advertisement text, without semantic filtering or field-specific restriction, making it impossible to separate cases where “Artificial Intelligence” is central to the position from those where it functions as a general, strategic or technical term. The initial database contained 800 jobs. Duplicate advertisements and jobs in languages other than English were deleted before their content was analyzed. At the same time, only the textual information in English was kept from the advertisements where the information was presented in both English and another language. After these procedures, the final database contained 797 jobs.

Before data analysis, several pre-processing and cleaning procedures were performed. Firstly, the database was transformed from a dataframe to a corpora (a collection of documents containing natural language text). The text step was the transformation of the corpus to a Document Term Matrix (DTM), which is a numerical representation of a text corpus in which each row corresponds to a document, each column to a unique term, and each cell indicates the frequency of that term in the document. In this stage, we also removed high-frequency words (such as connecting words and prepositions), symbols, punctuation marks, white spaces, numbers, and other high-frequency words (such as “intelligence,” “artificial,” “research,” “researchers,” etc.) from the text corpus. In this stage, we stemmed words manually by grouping them in a form with a common root or plural words into singular forms (for example, technologies became technology, technological became technology, models became model, and so on). Additionally, we transformed all words starting with capital letters to lowercase words. After these steps, we constructed visualizations such as word clouds on the top most frequent words in our job description. After this step, we processed the text by extracting bi-grams from the cleaned text, splitting them into individual words, and filtering for those occurring above a threshold of 35, for clearer and more meaningful visualization. These bi-grams were then transformed into a graph structure and visualized as a network to reveal common word pairings. For analysis and pre-processing, several R packages were used: tidytext, dplyr, igraph, ggraph, tm, topicmodels, and LDAtuning (Wickham et al., 2025; Silge and Robinson, 2016; Csárdi and Nepusz, 2006; Pedersen, 2024; Feinerer et al., 2008; Grün and Hornik, 2011; Nikita and Chaney, 2020).

**Figure 1:**
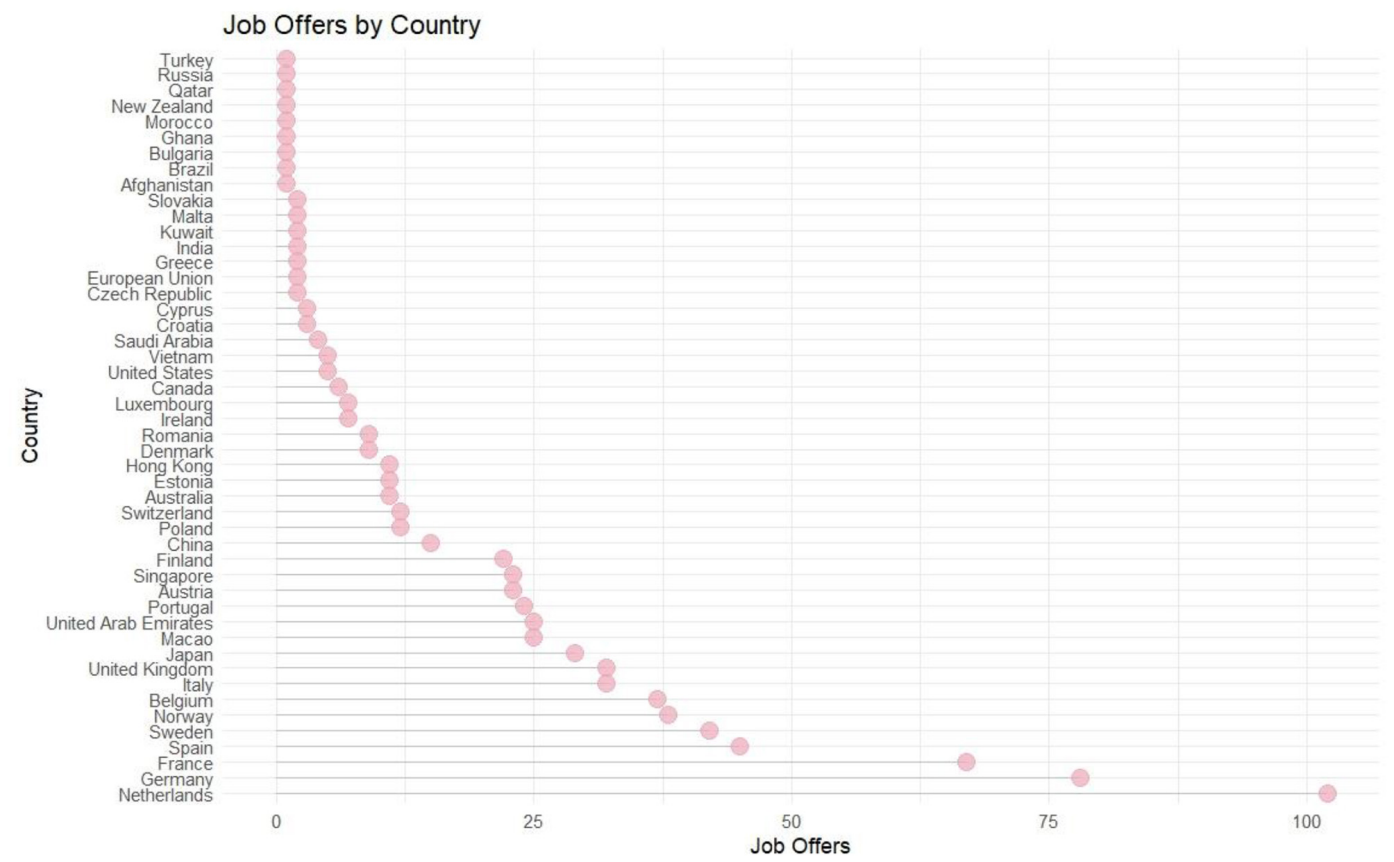
Jobs advertisements by country.

**Figure 2:**
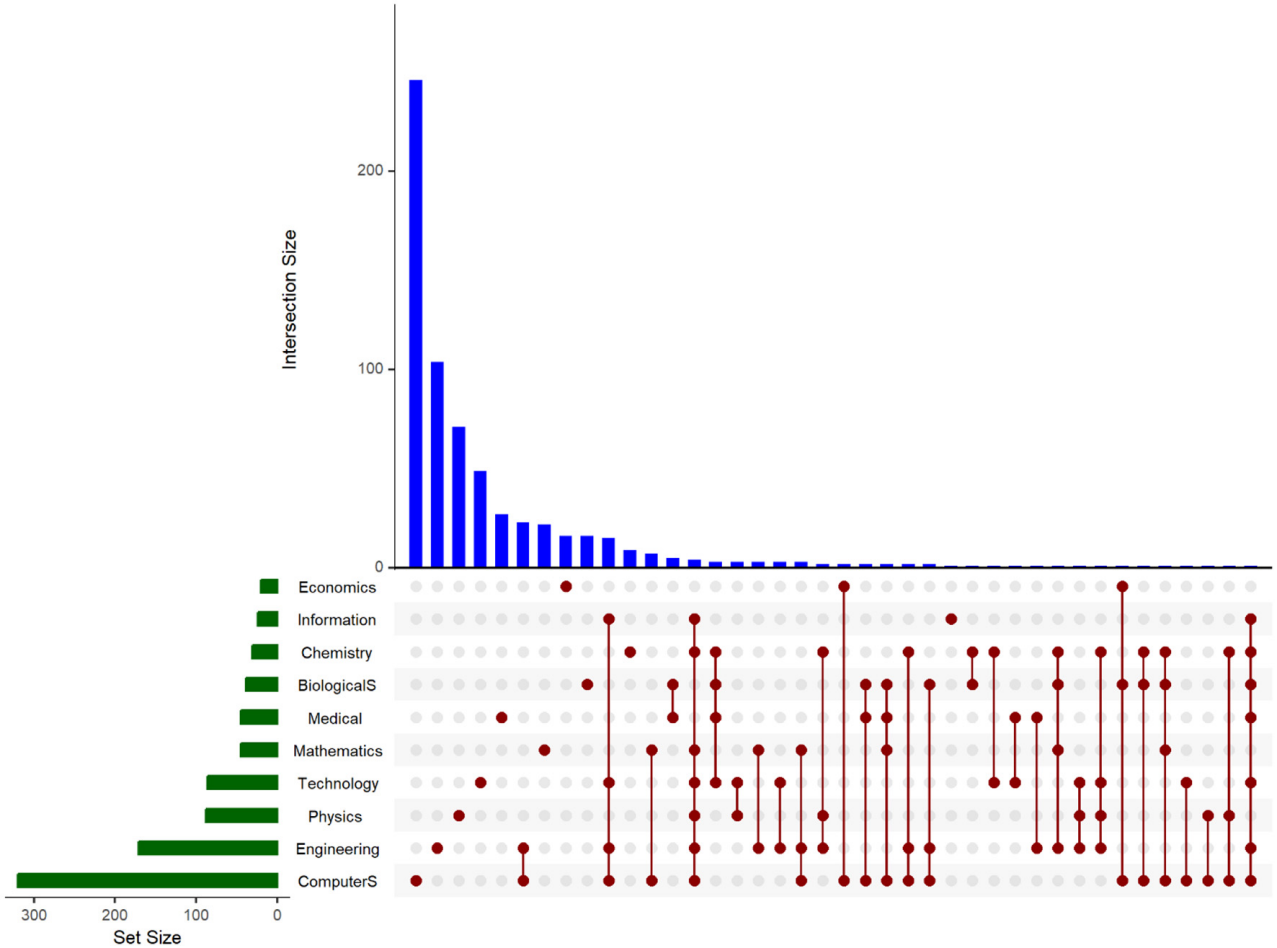
Jobs advertisements by research field.

Most job advertisements were posted by research institutions from the Netherlands (13%), Germany (10%), Germany (10%), France (8%), Spain (6%), Sweden (5%), Norway (5%), Belgium (5%), the United Kingdom (4%), Italy (4%) and Japan (4%; see Figure 1). At the same time, most job advertisements were for positions for early career researchers (Master's, PhD, or post-doctoral). On the other hand, most of the jobs posted were in the field of computer science (about 42% of the total advertisements were for this field, either in this field or in combination with other fields). We are use an UpSet plot (see Figure 2) to better understand the distribution of AI-related posts across research fields. This visualization approach enables us to emphasize the size of multiple sets and the intersections between them. In our investigation, the sets are the research fields to which each post belongs. The set size (green horizontal bars) shows that most job posts are in the Computer Science and Engineering fields. Economics and Information fields are not so well represented on the opposite pole. There are job posts that overlap between various combinations of fields. The blue vertical bars measure the intersection size for the sets indicated by the dark red dots. The set of job posts exclusively in Computer Science depicts the most significant dimension, showing that many research positions focus only on this domain. Our plot emphasizes some relevant situations, even if combinations on more than three fields have reduced frequencies. Job posts combining Computer Science and Engineering are more frequent, indicating their interconnection. Also, Technology and Information have a significant overlap with these two. Biological and Medical Sciences also depict relevant overlaps. Moreover, their overlap suggests the utility of Mathematics, Physics, and Technology for Engineering and Computer Science. While this data points to a high degree of interdisciplinarity, the UpSet plot also reveals a clear hierarchy. Fields like Computer Science and Engineering represent the dominant core, with other disciplines often appearing in combination with this technical base. This shows that although AI-related research is presented as collaborative, it is not necessarily a partnership of equals among fields, but one where other disciplines are frequently oriented around a computational center.

### 3.2 Method

Descriptive analyses were performed to see which competencies are most in demand among researchers in Artificial Intelligence or in disciplines that use (or deal with) artificial intelligence. Thus, word frequencies and frequencies of two-word combinations (bi-grams) were analyzed. By including bi-grams, we gain a better understanding of the corpus content. This means we treat pairs of adjacent words as individual units rather than analyzing words in isolation, which helps capture some of the syntactic relationships within the text. We used word cloud visual representations and a network representation to visualize the results.

Moreover, a topic modeling analysis using Latent Dirichlet Allocation (LDA) was used to extract relevant information about the types of jobs and the skills required. LDA is a probabilistic generative model aiming to reduce the size of large text data in order to reveal the thematic structure (Blei et al., 2003). The main assumptions of this technique are that (1) a text can be an amalgam of several topics, and these (2) topics are made up of several words with different probabilities of occurrence (Jelodar et al., 2019). Therefore, each document (in our study, a job advertisement) is defined by a multinomial distribution over a number of topics. Each of these topics should be understood as a multinomial distribution over the words in our DTM's vocabulary. In the literature, the document-topic probabilities are usually denoted by gamma. The purpose of the algorithm behind LDA is to estimate the parameters of these two distributions. The estimation methodology is based on Bayesian inference, using the Dirichlet distribution as a prior. A Markov Chain Monte Carlo (MCMC) algorithm, specifically Gibbs sampling, is used to compute the probability of a topic being represented by a certain term in the vocabulary. Consequently, this investigation allows us to understand which topics are relevant for the job advertisement collection and which terms best describe them. The LDA solution depends on the number of topics extracted. We based our decision on two criteria: one built on the standard cosine distance between two topics (Cao et al., 2009) and the other on the Jensen-Shannon divergence measure between all topics (Deveaud et al., 2014).

Text analysis methods, including topic modeling methods, have increased in popularity in various socio-economic research fields in recent years. The use of these methods in studies to estimate skill needs in the labor market is still in its early stages. There are, however, recent researchers who have utilized computational text analysis methods to examine competencies for research jobs among young people at the beginning of their careers (Maer-Matei et al., 2019; Mantai and Marrone, 2022), for jobs in data analytics or data science (Almgerbi et al., 2022; Radovilsky et al., 2018; Verma et al., 2019), for jobs in human resources (Chung and Chen, 2021), for jobs in marketing (Spada et al., 2022), for jobs in electrical engineering (Lyu and Liu, 2021) and so on.

## 4 Results

**Figure 3:**
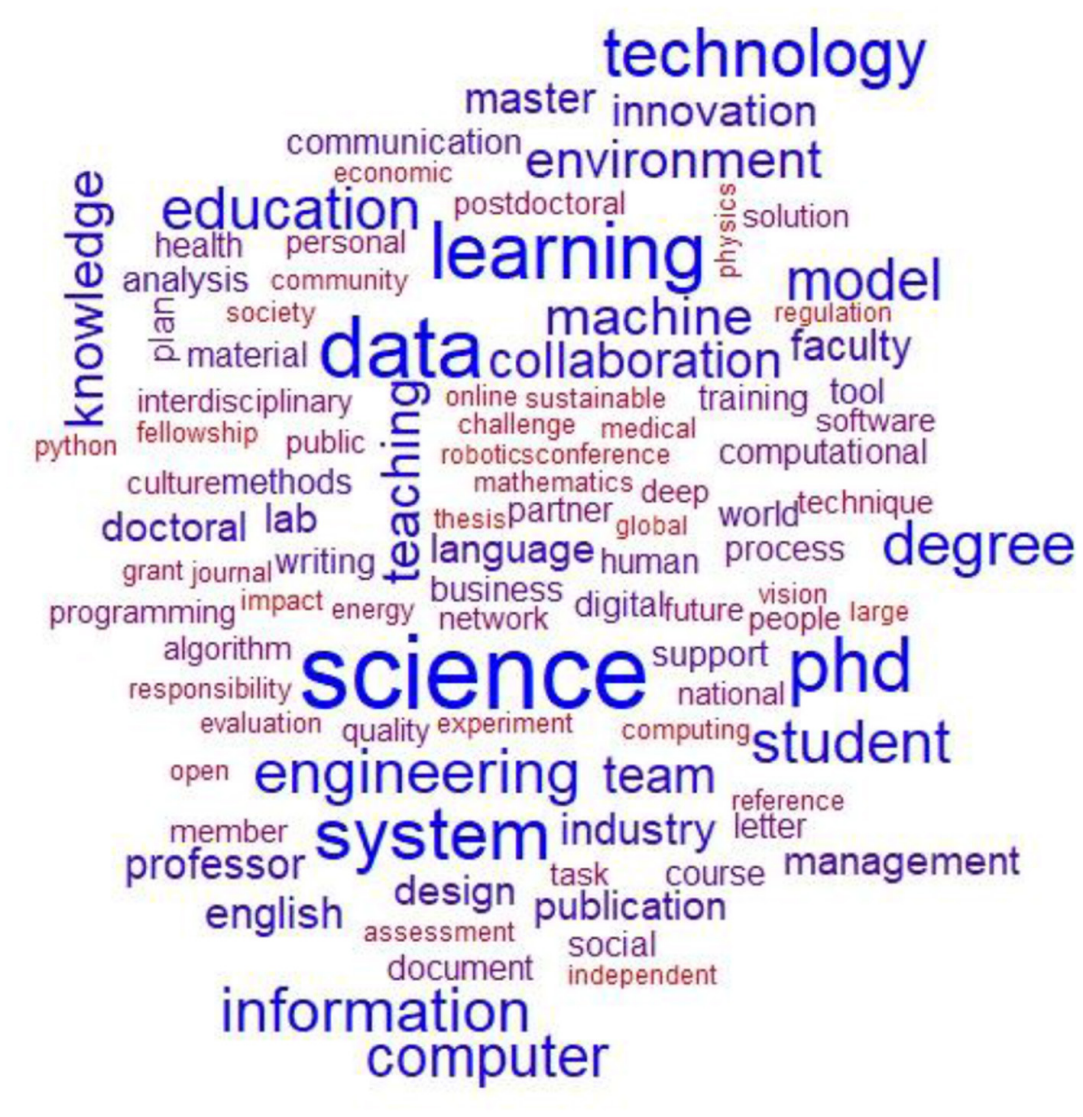
Word cloud with the most mentioned words in job advertisements.

**Figure 4:**
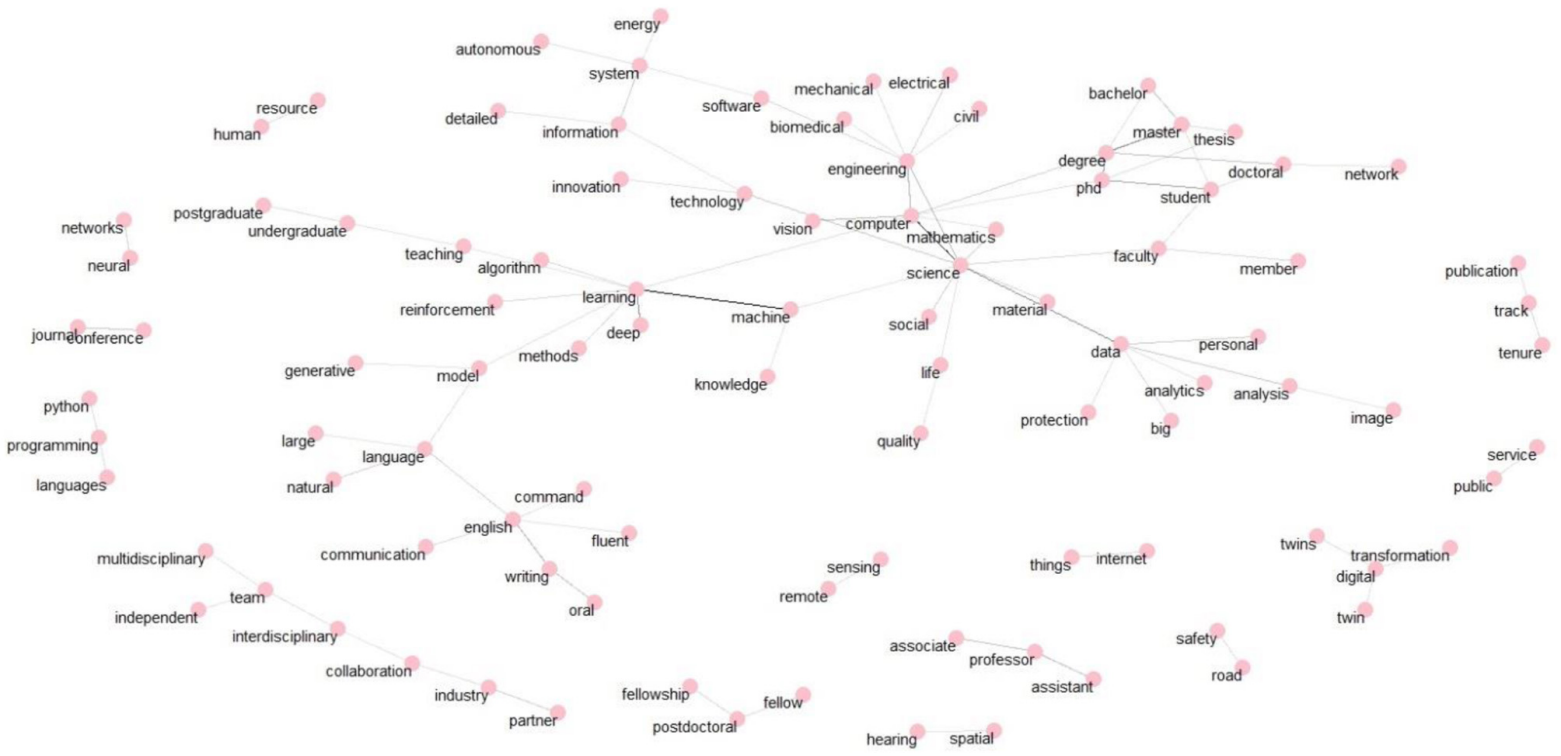
Most common bi-grams in job advertisements.

Figure 3 depicts a word cloud, where the size of each term reflects its frequency in the job postings. Figure 4 on the other hand shows a bi-gram analysis with the most common pairs of words appearing in the AI-related job advertisements. These two Figures depict the most salient skills and competencies in the job postings. An analysis of the most frequent words or combinations of two words (Figures 3, 4) in the advertisements indicates a strong presence of research jobs targeting certain topics or sub-domains in computer science, such as “*data science”* and “*machine learning*.” However, the words also indicate the presence of other STEM fields, such as engineering sciences, medicine, physics, and biology. Hence, when looking at the jobs posted about artificial intelligence epistemic culture, we can discern between different types of cultures or subcultures depending on the disciplinary focus of AI used as a tool for research. In other words, we can say that the AI-related academic jobs intersect with computational expertise and sector-specific knowledge. In this sense, these results confirm (Durette et al. 2014) regarding the importance of knowledge and specialized technical skills. As observed in previous research, it might also indicate, that artificial intelligence research has a strong interdisciplinarity and multidisciplinary nature (Hajkowicz et al., 2023).

Many of the words commonly found in advertisements for AI-related academic jobs are groups of skills identified by other researchers in the EURODOC report on identifying transferable skills and competencies to improve the employability and competitiveness of early-career researchers (Weber et al., 2018). A typology of skills in this report is those aimed at career development, such as building a curriculum vitae and writing a motivation/intention letter (which were removed from the corpus as they were present in all advertisements), as well as identifying potential referees/recommendations. The words targeting these skills are among the most frequent (“cv,” “reference,” “letter”) as they are the mandatory steps to complete the application for most jobs in this sector. These career development skills, such as CV preparation, motivation letters, and professional writing, are not only relevant for securing academic positions but also for broader career progression, especially for early career researchers (Okewole et al., 2020).

Another category of skills evident from the most common words in job advertisements is communication skills. Among the skills required in almost every research vacancy are academic communication and writing skills (especially English language skills, highlighted by the presence of the words: “English,” “writing,” and “English communication/command”) as well as academic writing and communication/presentation skills (“writing,” “conference,” “publication”). Communication and science dissemination skills are very important for both academic and public engagement (Brownell et al., 2013). These skills are in line with the increasing use of citizen-science and participatory approaches in many fields, especially in technically driven scientific activities, which can enhance transparency and foster public trust in scientific and technological developments (Năstasă et al., 2023). Another type of skill close to communication skills is inter-sectoral or international mobility competencies, which are evident from the texts analyzed through the knowledge of English or other languages of international circulation (“English,” “culture”). Another category concerns interpersonal skills highlighted by words referring to the ability to work and collaborate in a team (“team” and “collaboration”), independence (“independent”), responsibility (“responsibility”), and networking (“network”). Another category of skills is teaching and supervision (“teaching,” “course,” “supervision”). The literature also confirms the importance of teaching and supervision skills, becoming more and more relevant with the career progression (Mantai and Marrone, 2023; Maer-Matei et al., 2019).

Another category described by the EURODOC report refers to enterprise skills, which broadly refer to skills that help transfer knowledge to/from the economic environment. The most frequent words indicate innovation and industry collaboration as important elements. Moreover, words pertaining to digital skills typology are also evident, especially programming skills, and using and developing specific software (“programming,” “computational,” “digital,” “python,” “software”). These findings are consistent with previous results from (Maer-Matei et al. 2019) which signaled the importance of digital and technical skills especially data processing and analysis skills for early career researchers in STEM fields. Last but not least, another important category is research skills. These include methodological skills, data analysis skills, theoretical, interdisciplinary, ethics and academic integrity and grant writing skills (“data analysis,” “open,” “ethics,” “knowledge,” “interdisciplinary,” “evaluation,” etc.). The research skills, followed by digital skills, are among the most prevalent by words frequency in the job advertisements.

In addition to the typologies mentioned above, Figure 4 highlights technical and digital skills (“neural networks,” “phyton programming,” “natural language,” “internet (of) thing(s),” “reinforcement learning,” “deep learning,” “digital twin,” etc.). The most common bi-grams are the combinations “computer science” and “machine learning.” Furthermore, the most frequent word combinations are related to engineering sciences (“computer,” “software,” “civil,” “electrical,” “mechanical,” “biomedical,” etc.). Moreover, from these words, the names of the positions for which they apply (PhD student, postdoctoral student, professor, associate professor, and so on) are also evident. The associations around the term “data,” given by terms such as “big,” “analytics,” or “protection” illustrate the expertise required for data analysis and security. Once again, the interdisciplinary collaboration and the connection between academia and industry are underlined by the associations between terms like “multidisciplinary,” “interdisciplinary,” “collaboration” and “partner.” In summary, the requirements for AI-related research positions include technical skills, academic qualifications, effective communication and collaboration, and knowledge of emerging technologies. Our results are in line with previous works regarding the skills important in a research career, which showed the importance of research skills, digital skills, technical skills, communication skills, interpersonal skills, teaching and supervision skills, and enterprise skills (Mantai and Marrone, 2022, 2023).

**Figure 5:**
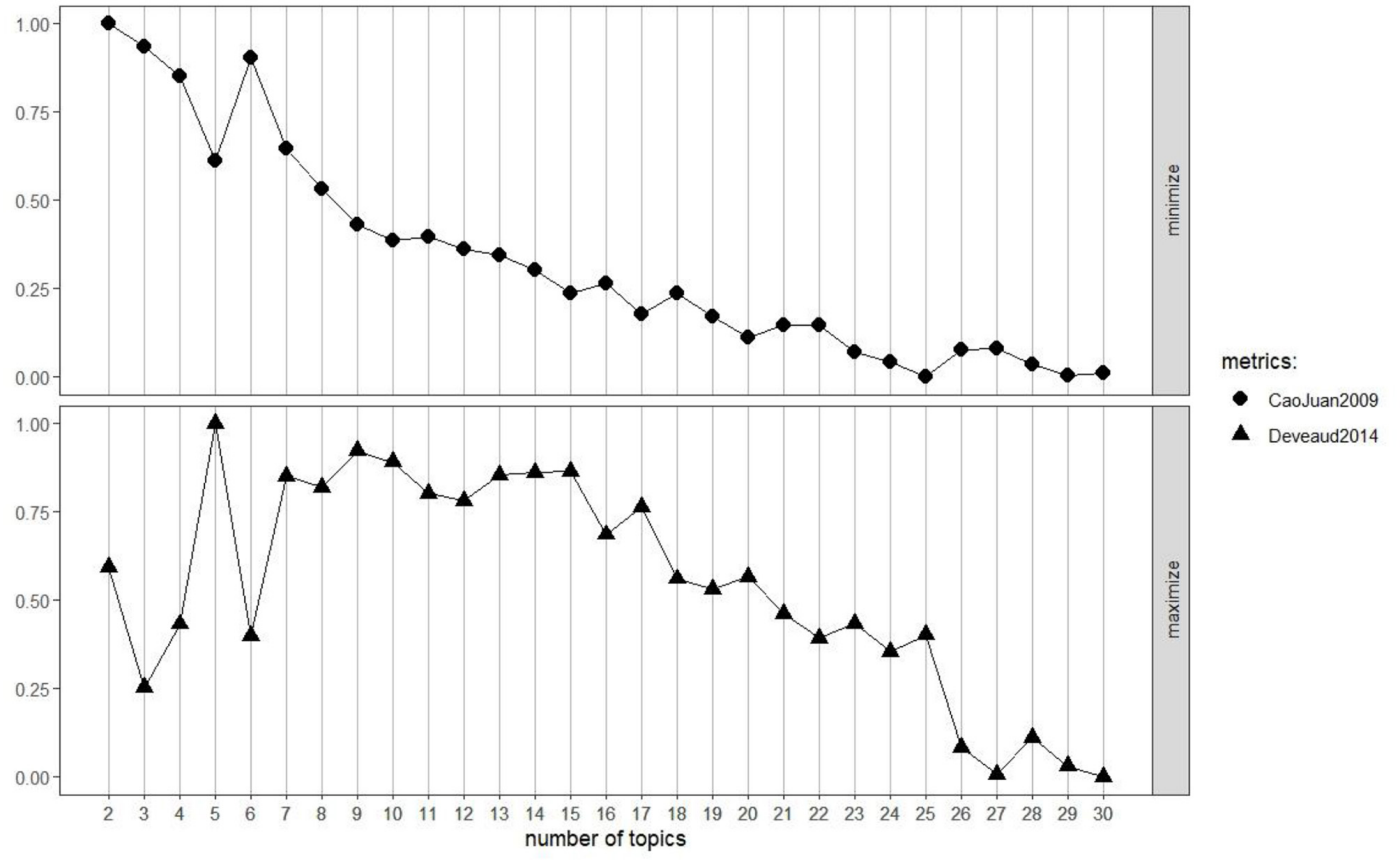
CaoJuan2009 and Daveud2014 measures for generating the optimal number of topics.

To analyze potential thematic and skill clusters in the jobs analyzed, we used topic modeling analysis based on the LDA algorithm. To generate the topics, it was necessary to transform the data into DTM format (the frequency of terms per document) and provide the number of topics to be generated. Conventionally, two measures are usually used to select the optimal number of topics, called Deveaud2014 and CaoJuan2009. These are used in combination with other criteria, such as the interpretability of topics. The Deveaud2014 measure (Deveaud et al., 2014) is based on maximizing the distances between topics, and the CaoJuan2009 measure (Cao et al., 2009) is based on minimizing the distances between topics. The results for the measures were not completely consistent (see Figure 5). The CaoJuan2009 measure indicated the optimal number of topics as 25, and the second indicated an optimal number of topics as 5. Under these circumstances, the most important criterion used for choosing the number of topics was the interpretability and consistency of the topics. After several attempts using 5 to 15 topics, we decided to go with the solution with 7 topics. This number was chosen because it offered the best balance between thematic detail and clarity; models with fewer topics (e.g., 5) tended to conflate distinct concepts into overly broad clusters, while models with a higher number of topics (e.g., 15) produced redundant themes that lacked clear interpretive distinction.

**Figure 6:**
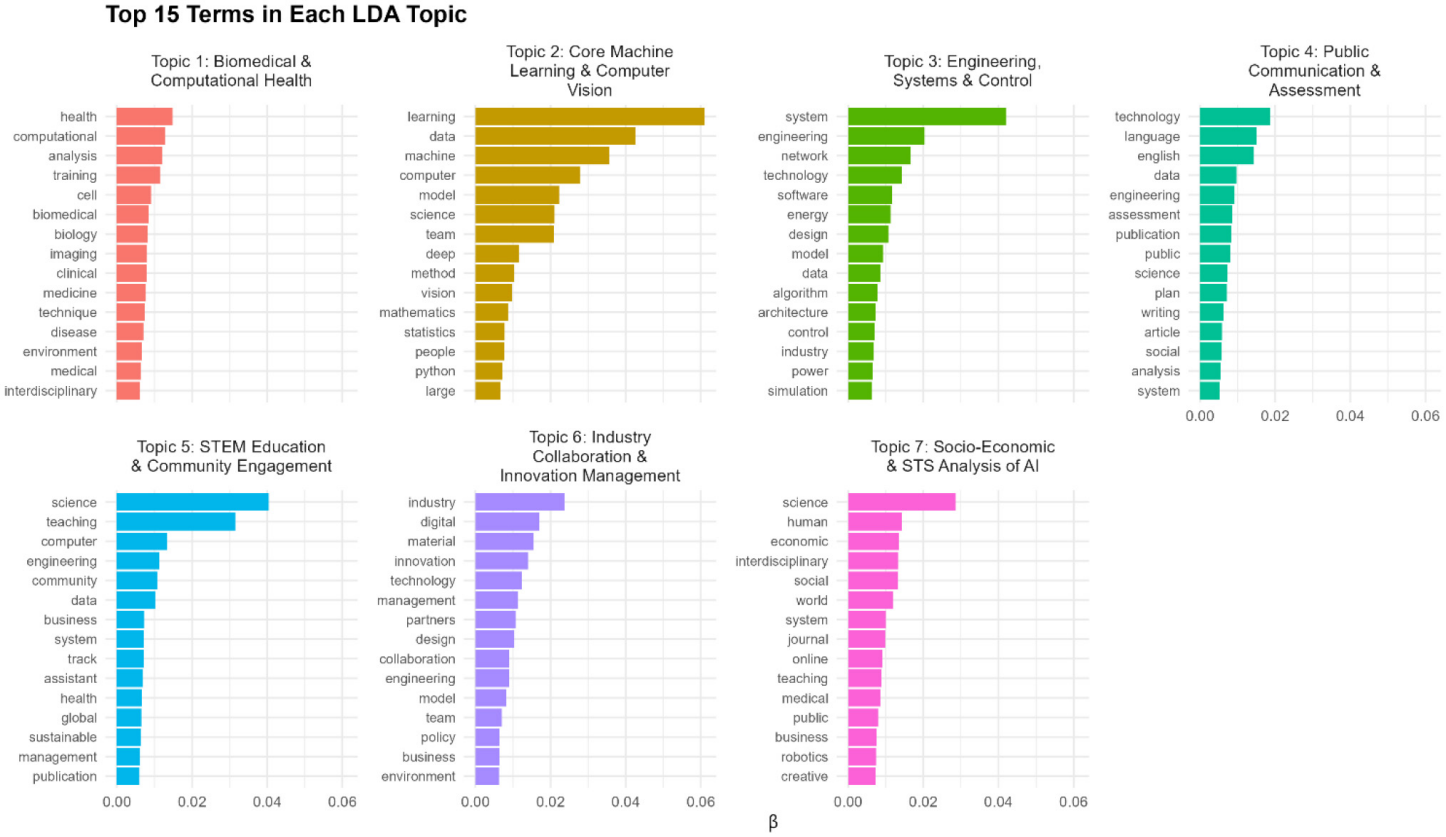
Top 15 words in the generated topics.

In the LDA results, the β (beta) values indicate the probability of each word belonging to a topic. Words with higher β values represent the most characteristic terms within that topic and thus provide insight into the thematic focus of the cluster. In addition to the frequency-based analyses, the LDA topic modeling groups these skills and competencies into distinct thematic orientations. These topics emphasize distinct epistemic cultures (See Figure 6). These are differentiated in how knowledge is produced, validated, and socially organized within scientific and professional communities. The first topic is heavily centered on a formal-technical epistemic culture, prioritizing mathematical modeling, computational efficiency, and reproducibility. While the second topic also focuses on models and algorithms for validating knowledge, it also implies interdisciplinarity and a culture focused on ethical constraints. The third topic reflects a design-based epistemic culture, where functionality and real-world applications are of great importance. The fourth and fifth topics emphasize a culture focused on pedagogy and public dissemination. The next topic implies an entrepreneurial epistemic culture, where knowledge is judged by its relevance to the market and profits. Finally, a socio-economic and STS-oriented culture is more critical because it analyzes the societal implications, ethical dimensions, and power structures related to AI systems. These topics indicate different but intertwined epistemic cultures, showing that AI-related research is not a single culture, but a field shaped by multiple logics and focuses of knowledge-making.

A more in-depth description of Figure 6 indicates that the first topic is a grouping of words related to jobs in health and biomedicine that use computational artificial intelligence tools. The skills in this topic cover computational methods, experience with imaging data and cross-disciplinary knowledge (for example, computer science, biology, and the medical field). Moreover, this topic may also imply ethical and regulatory literacy, as it deals often with data on human subjects. The second topic concerns jobs related to core machine learning and AI-related research. The skills covered by this topic imply programming skills (machine learning, deep learning, computer vision, Python programming), knowledge of foundational mathematics/statistics, and interpersonal skills are required. This topic also emerges in another study focused on industry AI-related vacancies by (Borgonovi et al. 2023) as a top sought-after category of skills. The third topic covers jobs in engineering sciences, particularly those related to system/network architecture. The words mainly indicate a demand for digital programming skills (knowledge of methods and algorithms) applied in engineering, industrial control, and energy systems. There is an emphasis on systems, networks, software engineering, simulation, and power/control. The fourth topic emphasizes roles at the interface of AI research and public communication. It covers skills related to communication, language, and dissemination. The fifth topic covers jobs that place a strong emphasis on teaching skills in engineering and computer science. It is focused on STEM education, community engagement, and sustainable/global programs. The sixth topic is about industry collaboration and innovation management. It covers jobs that require “enterprise” skills for the transfer of knowledge to the business and industry environment. Previous research examining online vacancies in OECD countries depicted a similar cluster of skills as important for the AI field (Borgonovi et al., 2023). It is most likely a topic that represents an intersection between AI, industry collaboration, innovation policy, and business partnerships. Last but not least, the final topic offers a concrete example of the hybrid epistemic cultures, showing interdisciplinary jobs where socio-economic sciences intersect with artificial intelligence. This topic, which involves roles in fields like Science and Technology Studies (STS), signals a demand for researchers who can bridge the technical and the social, a skill set fundamentally different from the more purely computational or engineering-focused roles identified in other clusters.

**Figure 7:**
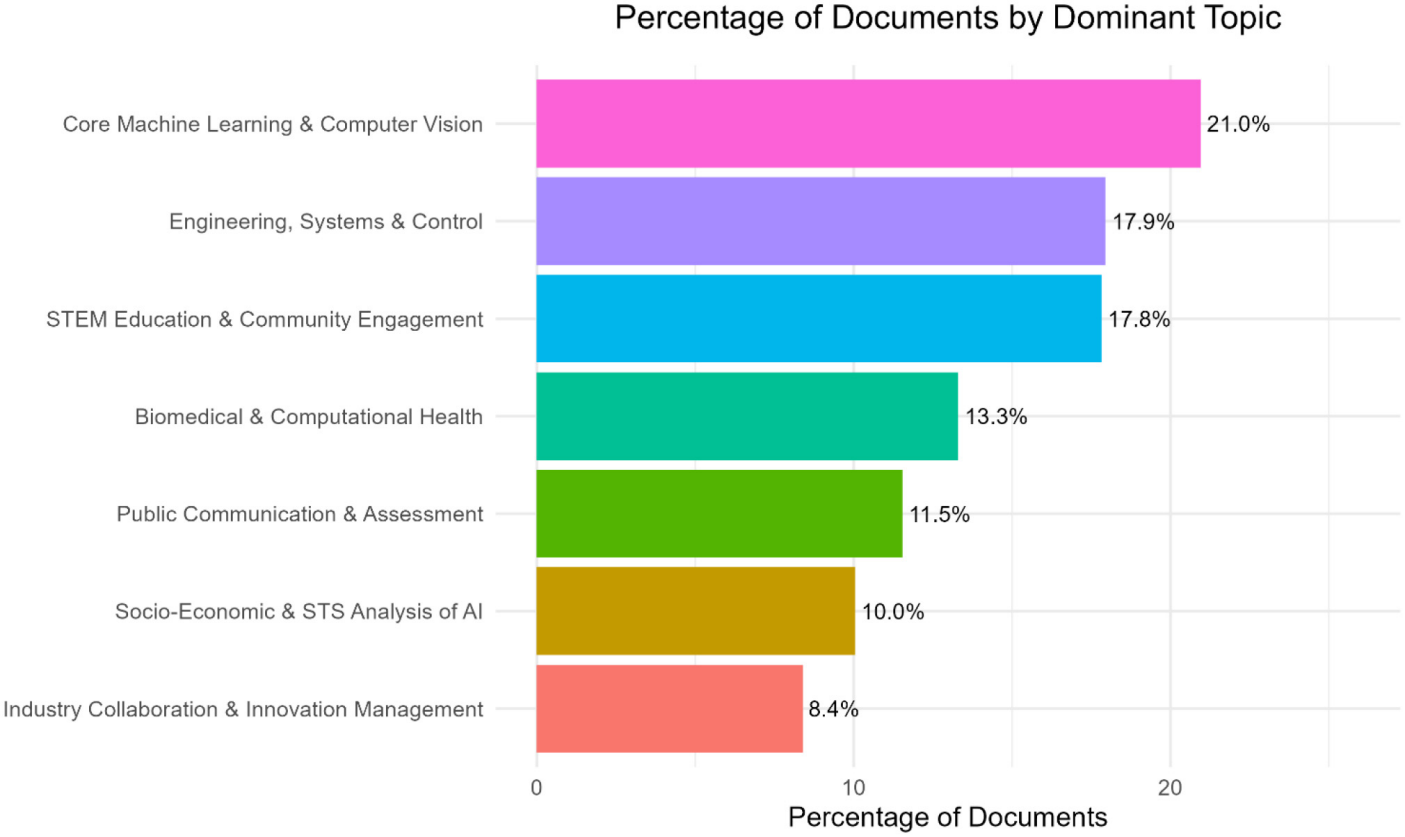
Distribution of Dominant Topics in AI-related academic jobs (%).

Analysis of the percentage of documents by the dominant topic, as shown by the document–topic proportions (gamma probabilities; see Figure 7), indicates that Topic 2 (Core Machine Learning and Computer Vision) is the dominant topic across the dataset, accounting for 21.0% of all documents. The next predominant theme among the documents is Topic 3 (Engineering, Systems and Control) with 17.9% and Topic 5 (STEM Education and Community Engagement) with 17.8%. The other topics have smaller shares, with Industry Collaboration and Innovation Management (Topic 6) being the least dominant at 8.4%. This distribution confirms that the technical core of AI, particularly machine learning and computer vision, is central in the job advertisements. At the same time, the other topics are secondary but still play notable roles (Figure 7).

## 5 Conclusions and discussions

Our research explored the skills and competencies prioritized in academic job postings that fall under the umbrella of 'artificial intelligence' and related fields. The findings show, as expected, an emphasis on skills required in a series of STEM (science, technology, engineering, and mathematics) domains that use Artificial Intelligence, such as computer science, engineering, medicine, physics, and biology. Interestingly, as they are represented in these job advertisements, our findings suggest that the AI-related research is portrayed as being characterized by multiple epistemic cultures rather than a single, uniform culture. This portrayal presents a field deeply interconnected with interdisciplinary and multidisciplinary approaches, where different sub-communities signal distinct required competencies to prospective researchers.

Our analysis also showed the importance of research skills such as methodological proficiency, data analysis, interdisciplinary knowledge, ethics, and grant writing. Digital and technical skills, such as programming and software development, are also important. Other groups of skills include communication skills (e.g., academic writing, English proficiency), interpersonal skills (e.g., teamwork, collaboration), teaching and supervision capabilities, and enterprise skills (innovation and industry knowledge transfer). The results are consistent with previous findings (Mantai and Marrone, 2022; Weber et al., 2018; Mantai and Marrone, 2023).

Unlike previous studies focusing primarily on general labor market skills or skills needed by early-career researchers across various domains, our analysis targets AI-related academic positions and systematically maps the diverse skills highlighted in job advertisements. Additionally, our application of text mining techniques, including topic modeling, allows for a visualization of the distinct thematic clusters that represent these different epistemic orientations, highlighting how both technical and interdisciplinary skill sets are discursively constructed. By applying a novel methodological approach in the study of public presentation and justification of epistemic cultures, using data from an academic job board and a text mining analytical approach, we enrich current discussions by explicitly linking required competencies to underlying epistemic frameworks.

Our analysis indicates the possible emergence of new hybrid epistemic cultures within AI-related research, characterized by skills that overlap multiple disciplinary traditions, such as computational methods, communication, and interdisciplinary collaboration. These hybrid cultures may influence how knowledge is validated, and innovation occurs, representing an important direction for future investigation.

Additionally, the varying emphasis placed on specialized technical skills vs. more generic competencies, such as communication and enterprise skills, raises questions about their impact on career trajectories and research productivity. Future studies could explore how these skill priorities shape research communities, their outputs, and interdisciplinary practices within artificial intelligence.

The findings are valuable for learners and students interested in pursuing an AI-related academic career. The overview can provide a starting point for students at the upper secondary, bachelor's, or master's level on what skills, knowledge, specific tools, and qualifications are needed for research jobs in this area. Additionally, the terms identified in our analysis may highlight underexplored areas where gaps exist, attracting prospective researchers.

These results can be useful for educators, graduate schools, universities, as well as other types of research institutions or companies to adapt their curricula and their educational offers to target these in-demand skills for AI-related academic positions. On the same line, as previous authors noted, some skills are not well described and tackled in Ph.D. programs' description but are reflected in job advertisements (Borrell-Damian et al., 2015; Mantai and Marrone, 2022). Hence, comprehensive descriptions of artificial intelligence educational programs to capture soft and hard skills are highly needed.

Additionally, the results can inform policymakers in making appropriate education and research funding decisions. These specific areas and skills identified in the current research can guide funding allocation to develop skills currently needed and support scarcely addressed research relevant to ongoing technological changes. For example, interdisciplinary projects and educational programs that address social risks, ethical implications, and surveillance are highly needed in light of current technological innovations.

Our research presents some limitations. For instance, we analyze job advertisements from academic institutions such as universities or research institutes posted on Euraxess. However, these job advertisements do not provide an exhaustive view, as we did not include AI-related job postings from companies. This exclusion might limit a comprehensive understanding of current AI-related skills demands, especially considering that significant innovations are emerging from private companies (such as OpenAI, Anthropic, Alphabet etc.) and university-industry partnerships. Exploring job postings from other sources may generate interesting insights. In future research, we will analyze more jobs from different types of organizations in order to address this limitation.

Another limitation is that the present research provides an exploratory, descriptive view of the skills needed in AI-related academic jobs. In future research directions, we plan to complement these computational methods with in-depth qualitative analysis, such as interviews with hiring managers and researchers, to provide a richer, more grounded view of the skills and epistemic cultures in AI-related academic jobs.

This study shows the power of analyzing job advertisements not merely as lists of requirements, but as the discursive “vocabularies of motive” that actively construct the identity of the sought-after researcher. The diverse and sometimes competing skill sets revealed in these postings show that the field of “Artificial Intelligence” is not a uniform entity but a collection of emerging epistemic cultures. As these hybrid cultures continue to evolve, blending deep technical expertise with social, ethical, and entrepreneurial competencies, the language used to recruit the next generation of researchers will not only reflect the state of the field but will shape its future trajectory and social impact.

## Data Availability

The raw data supporting the conclusions of this article will be made available by the authors, without undue reservation.
